# High Dose Lopinavir/Ritonavir Does Not Lead to Sufficient Plasma Levels to Inhibit SARS-CoV-2 in Hospitalized Patients With COVID-19

**DOI:** 10.3389/fphar.2021.704767

**Published:** 2021-07-01

**Authors:** Mario Karolyi, Sara Omid, Erich Pawelka, Bernd Jilma, Thomas Stimpfl, Christian Schoergenhofer, Hermann Laferl, Tamara Seitz, Marianna Traugott, Christoph Wenisch, Alexander Zoufaly

**Affiliations:** ^1^ Department for Infectious Diseases and Tropical Medicine, Klinik Favoriten, Vienna, Austria; ^2^ Department of Clinical Pharmacology, Medical University of Vienna, Vienna, Austria; ^3^ Department of Laboratory Medicine, Medical University of Vienna, Vienna, Austria

**Keywords:** treatment, severe COVID-19, plasma concentration, antivirals, lopinavir, ritonavir

## Abstract

**Background:** Despite lopinavir/ritonavir (LPV/RTV) demonstrating *in-vitro* activity against SARS-CoV-2, large trials failed to show any net clinical benefit. Since SARS-CoV-2 has an EC50 of 16.4 μg/ml for LPV this could be due to inadequate dosing.

**Methods:** COVID-19 positive patients admitted to the hospital who received high dose LPV/RTV were included. High dose (HD) LPV/RTV 200/50 mg was defined as four tablets bid as loading dose, then three tablets bid for up to 10 days. Trough plasma concentrations were measured after the loading dose and on day 5–7 in steady state (SS). Post loading dose (PLD) and SS plasma trough levels were compared with SS trough levels from COVID-19 patients who received normal dose (ND) LPV/RTV (2 tablets bid) at the beginning of the pandemic.

**Results:** Fifty patients (30% female) with a median age of 59 years (interquartile range 49–70.25) received HD LPV/RTV. Median HD-PLD concentration was 24.9 μg/ml (IQR 15.8–30.3) and significantly higher than HD-SS (12.9 μg/ml, IQR 7.2–19.5, *p* < 0.001) and ND-SS (13.6 μg/ml, IQR 10.1–22.2, *p* = 0.013). HD-SS and ND-SS plasma levels did not differ significantly (*p* = 0.507). C-reactive-protein showed a positive correlation with HD-SS (Spearman correlation-coefficient rS = 0.42, *p* = 0.014) and ND-SS (rS = 0.81, *p* = 0.015) but not with HD-PLD (rS = 0.123, *p* = 0.43).

**Conclusion:** HD-PLD plasma trough concentration was significantly higher than HD-SS and ND-SS concentration, but no difference was detected between HD-SS and ND-SS trough levels. Due to the high EC50 of SARS-CoV-2 and the fact that LPV/RTV is highly protein bound, it seems unlikely that LPV/RTV exhibits a relevant antiviral effect against SARS-CoV-2 *in vivo*.

## Introduction

The human immunodeficiency virus (HIV) drug lopinavir/ritonavir (LPV/RTV) has shown *in-vitro* efficacy in SARS, MERS and SARS-CoV-2 and appeared to be a promising drug at the beginning of the COVID-19 pandemic (Choy et al., 2020; Sanders et al., 2020). The first randomized controlled trial investigating the potential of LPV/RTV in approximately 200 COVID-19 patients was the LOTUS trial, which did not show any effect on time to clinical recovery or 28-days mortality (Cao et al., 2020). The RECOVERY trial where 1,616 and 3,424 patients received LPV/RTV and standard of care respectively, did not demonstrate any beneficial effects on 28-days mortality, length of stay or disease progression (Collaborative Gr, 2020). The results from the SOLIDARITY trial confirmed the former results; no reduction in 28-days mortality could be shown with administration of LPV/RTV (Pan et al., 2021).

One reason for the lack of any clinical benefit could be inadequate dosing of LPV/RTV, because the above-mentioned studies used the standard dosing regimen for LPV/RTV (200/50 mg two tablets bid) as used in HIV patients (Cao et al., 2020; Collaborative Gr, 2020; Pan et al., 2021). In a small case series of eight patients, we could show the median LPV steady state plasma concentration in COVID-19 patients was 13.6 μg/ml (Schoergenhofer et al., 2020) and below the EC50 of 16.4 μg/ml for SARS-CoV-2 (Choy et al., 2020).

In the ongoing Austrian Coronavirus Adaptive Clinical Trial (ACOVACT) (NCT04351724) we are using a high dosing scheme consisting of a loading dose of four tablets bid on day 1 followed by three tablets bid for up to 10 days (NCT04351724). The results of the above-mentioned studies led us to increase the dose of LPV/RTV. The aim of our study was to evaluate if a higher dosing scheme would result in sufficient plasma levels to potentially inhibit SARS-CoV-2 replication. We measured LPV/RTV trough plasma levels in our patients after the loading dose and during steady state to evaluate if this dosing scheme would result in sufficient plasma levels. Furthermore we compared the plasma levels of high dose (HD) LPV/RTV with COVID-19 patients who received the normal dose (ND) LPV/RTV in the beginning of the pandemic.

## Methods

### Study Design and Population

Data from the high dose group was collected as part of the ongoing Austrian Coronavirus Adaptive Clinical Trial (ACOVACT) (NCT04351724). In this trial patients ≥18 years of age with clinical signs and symptoms of respiratory tract infection and molecular confirmed SARS-CoV-2 infection, need for hospitalization and oxygen saturation <94% w/o oxygen insufflation or >3% drop of oxygen saturation in case of chronic obstructive lung disease were randomized to either high dose (HD) LPV/RTV or standard of care. The diagnostic work-up included medical history of COVID-19 symptoms (e.g., fever, headache, cough, dyspnea, loss of smell), clinical status and chest X-ray. Only symptomatic patients with molecular confirmed SARS-CoV-2 infection were included in our study.

For this study, data from patients who were randomized to the HD LPV/RTV group and who had a least one of the two planned plasma concentrations measured were eligible. The first plasma trough concentration was taken after the loading dose, prior to administration of the maintenance dose, and is termed the post loading dose (PLD) throughout the text. The second trough level was taken in steady state (SS) between days 5 and 7 of treatment, immediately prior to administration of the next dose.

Patients in the HD LPV/RTV group received four tablets (200/50 mg) bid as a loading dose on day 1 and 3 tablets bid for up to 10 days. The patients received the study drug and all of their medication on a daily basis by the nurses during their hospital stay. All patients signed an informed consent form.

At the beginning of the COVID-19 pandemic we used the LPV/RTV two tablets bid as an off-label therapy if patients agreed to that treatment. Only symptomatic patients with molecular proven SARS-CoV-2 infection were included in this group as well. During this period, we were able to collect plasma trough SS concentration from eight patients, termed normal dose steady state (ND-SS) throughout the text.

HD-PLD, HD-SS and ND-SS plasma trough levels were compared statistically in our study.

### LPV/RTV Measurements

Analyses of plasma trough concentrations for LPV and RTV were performed at the Department of Laboratory Medicine of the Medical University of Vienna. After blood samples had been drawn, they were either sent directly to the laboratory or were centrifuged, frozen at −20°C at our department, and transferred in batches.

Quantitative measurements of total LPV and RTV concentrations were performed by liquid chromatography–tandem mass spectrometry (LC-MS/MS) using the IVD-CE certified Assay MassTox® TDM (Chromsystems Instruments and Chemicals GmbH, Gräfeling, Germany). The lower limit of quantification of LPV and RTV was 0.732 μg/ml and 0.189 μg/ml, respectively.

### Statistical Analysis and Data Collection

Data was entered in a MS Excel sheet (Microsoft, Redmond, WA, United States) and anonymized before statistical analysis. All analyses were made with SPSS 25 (IBM, Armonk, NY, United States) for Mac OS (Apple, Cupertino, CA, United States). Categorial variables were described as counts and percentages. For metric, non-normally distributed variables the median (Md) and interquartile range (IQR) was used. Significance tests for categorial variables were made via cross-tables and Chi^2^ tests or Fisher-exact where applicable. To compare the matched samples of HD-PLD and HD-SS Wilcoxon signed-rank test was used. To compare the differences between HD and ND groups the Mann-Whitney-U-test was used. Spearman rank correlation coefficient was used to reduce the effect of any outliers on the results. A two-sided alpha <0.05 was considered statistically significant.

## Results

### Demographics

Plasma samples from 50 patients who received high dose LPV/RTV were available. Post-loading dose drug levels (PLD), steady state drug levels (SS) or both were available in 43, 33, and 26 patients respectively. The SS trough levels were taken on treatment day 6 (IQR 5–6).

The median age of the patients was 59 years (IQR 49–70.25) and 30% were female. Median time from symptom onset to treatment initiation was 7 days (IQR 5–9.25). The three most common comorbidities were hypertension (50%), diabetes mellitus type 2 (24%) and coronary artery disease (14%). Eight patients (16%) had to be transferred to the ICU and two patients (4%) died during their hospital stay. Most patients (80%) in the HD group received dexamethasone as an anti-inflammatory treatment.

The normal dose (ND) group consisted of eight patients (62.5% female) and had a median age of 59 years (IQR 32.5–70.75). SS ND trough levels were taken on day 5 (IQR 4–9.25). No patients in this group received dexamethasone treatment. For details see Table 1.

**TABLE 1:** Patients characteristics.

	High dose	Normal dose	*p*-value
Sex female	15/50 (30%)	5/8 (62.5%)	0.110
Age in years (Md, IQR)	59 (49–70.25)	59 (32.5–70.75)	0.830
BMI (Md, IQR)	30 (27.8–32.3)	28.5 (24.5–30.3) *N* = 7	0.171
Hypertension	25/50 (50%)	Missing	
Diabetes mellitus type 2	12/50 (24%)	Missing	
Coronary artery disease	7/50 (14%)	Missing	
COPD	5/50 (10%)	Missing	
Chronic kidney disease	4/50 (8%)	Missing	
Atrial fibrillation	3/50 (6%)	Missing	
ICU admission	8/50 (16%)	0/8 (0%)	0.583
In-hospital mortality	2/50 (4%)	0/8 (0%)	1.0
Time from symptom onset to treatment in days (Md, IQR)	7 (5–9.25)	Missing	
SS treatment day (Md, IQR)	6 (5–6) *n* = 33	5 (4–9.25) *n* = 8	0.784
Dexamethasone	40/50 (80%)	0/8 (0%)	<0.001

BMI, body-mass-index; COPD, chronic obstructive pulmonary disease; ICU, intensive care unit; IQR, interquartile range; Md, Median; SS, steady state.

The administration of dexamethasone as standard of care was implemented throughout the pandemic because results of RECOVERY dexamethasone trial were published (Horby et al., 2021). As patients in the normal dose group were included early on in the pandemic, they did not receive dexamethasone treatment. No patients received IL-6 blocking agents.

### LPV/RTV Plasma Concentration

**FIGURE 1:**
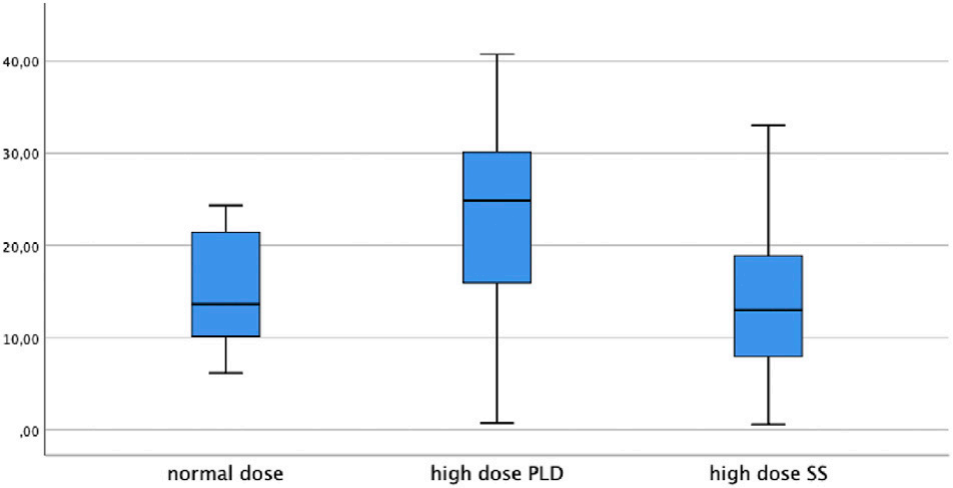
LPV plasma trough levels in μg/ml.

LPV trough levels were significantly higher post-loading dose compared to steady state (median 24.9 μg/ml, IQR 15.8–30.3 and 12.9 μg/ml, IQR 7.2–19.5 respectively, *p* < 0.001). Median ND-SS trough level was 13.6 μg/ml (IQR 10.1–22.2) and was significantly lower than the HD-PLD (*p* = 0.013). In contrast, the ND-SS and HD-SS did not differ significantly (*p* = 0.507). The LPV concentrations are depicted in Figure 1.

RTV median HD-PLD and SS trough levels were 1.2 μg/ml (IQR (0.6–1.7) and 0.22 μg/ml (IQR 0.19–0.74) respectively and thus significantly higher (*p* < 0.001). Median ND-SS trough level was 0.19 μg/ml (IQR 0.19–0.48) and was significantly lower than the HD-PLD (*p* < 0.001). In contrast the ND-SS and HD-SS did not differ significantly (*p* = 0.961). For details see Table 2.

**TABLE 2:** Plasma concentrations.

	High dose PLD *n* = 43	High dose SS (hdSS) *n* = 33	*p*-value PLD-hdSS *n* = 26	Normal dose SS (ndSS) *n* = 8	*p*-value PLD-ndSS *n* = 51	*p*-value hdSS-ndSS *n* = 41
LPV	24.9 μg/ml (IQR 15.8–30.3)	12.9 μg/ml (IQR 7.2–19.5)	<0.001	13.6 μg/ml (IQR 10.1–22.2)	0.013	0.507
RTV	1.2 μg/ml (IQR 0.6–1.7)	0.22 μg/ml (IQR 0.19–0.74)	<0.001	0.19 μg/ml (IQR 0.19–0.48)	<0.001	0.961

IQR, interquartile range; LPV, lopinavir; PLD, post loading dose; RTV, ritonavir; SS, steady state.

### Side Effects in the High Dose LPV/RTV Group

Nine (18%) patients developed diarrhea and two (4%) patients complained of nausea. An increase in alanine-amino-transferase (ALAT) of ≥3 times the upper limit of normal (ULN) and ≥5 times the ULN was observed in 12 (24%) and 6 (12%) of the patients respectively.

Patients with LPV PLD or LPV SS above the median did not have a significantly higher incidence of diarrhea, nausea or ALAT ≥3 ULN. An increase of ALAT ≥5 ULN was significantly more frequent in patients who had a LPV PLD above the median (27.3 vs. 0% in patients below the median, *p* = 0.021). For details see Table 3.

**TABLE 3:** Side effects of patients receiving high dose LPV/RTV.

	Total	LPV PLD < Md	LPV PLD > Md	*p*-value	LPV SS < Md	LPV SS > Md	*p*-value
Diarrhea	9/50 (18%)	5/21 (23.8%)	4/22 (18.2%)	0.721	3/16 (18.8%)	2/17 (11.8%)	0.656
Nausea	2/50 (4%)	0/21 (0%)	2/22 (9.2%)	0.488	0/16 (0%)	1/17 (5.9%)	1.0
ALAT ≥ 3ULN	12/50 (24%)	3/21 (14.3%)	8/22 (36.4%)	0.162	4/16 (25%)	5/17 (29.4%)	1.0
ALAT ≥ 5ULN	6/50 (12%)	0/21 (0%)	6/22 (27.3%)	0.021	2/16 (12.5%)	2/17 (11.8%)	1.0

ALAT, Alanine-amino-transferase; LPV, lopinavir; Md, Median; PLD, post loading dose; SS, steady state; ULN, upper limit of normal.

The side effects in the ND group were not monitored.

### Gender Differences and Correlations

Gender differences were only analyzed in the high dose LPV/RTV group. There was a non-significant trend towards a higher LPV PLD in female compared to male patients, with median plasma levels of 29.1 μg/ml (IQR 20.7–31) and 23.1 μg/ml (13.6–29.03) respectively (*p* = 0.052). Gender did not affect the LPV SS plasma trough levels (*p* = 0.488).

RTV PLD was significantly higher in female patients (*p* = 0.013) but no difference was observed in the RTV SS plasma levels (*p* = 0.528), as can be seen in Table 4.

**TABLE 4:** Gender differences of patients receiving high dose LPV/RTV.

	Male	Female	*p*-value
LPV PLD	23.1 μg/ml (IQR 13.6–29.0) *n* = 29	29.1 μg/ml (IQR 20.7–31) *n* = 14	0.052
RTV PLD	0.94 μg/ml (IQR 0.59–1.48) *n* = 29	1.46 μg/ml (IQR 1.2–2.3) *n* = 14	0.013
LPV SS	12.1 μg/ml (IQR 6.9–18.7) *n* = 25	15.6 μg/ml (IQR 6.9–22.3) *n* = 8	0.488
RTV SS	0.21 μg/ml (IQR 0.19–0.70) *n* = 25	0.46 μg/ml (IQR 0.16–1.43) *n* = 8	0.528

IQR, interquartile range; LPV, lopinavir; PLD, post loading dose; RTV, ritonavir; SS, steady state.

Median C-reactive-protein (CRP) levels were 58 mg/l (IQR 24–106), 8.3 mg/l (IQR 3–65.9) and 16.6 mg/l (IQR 4.9–39.8) in the HD-PLD, HD-SS and ND-SS group respectively. CRP levels were significantly higher in the HD-PLD group.

Furthermore, CRP showed a positive correlation with HD-SS (Spearman rank correlation coefficient rS = 0.42, *p* = 0.014) and ND-SS (rS = 0.81, *p* = 0.015) but not with HD-PLD (rS = 0.123, *p* = 0.43).

## Discussion

In patients with moderate to severe COVID-19, a lopinavir/ritonavir loading dose of four tablets bid increased the lopinavir plasma levels well above the EC50 for SARS-CoV-2 (Choy et al., 2020) during the first days of treatment, not considering protein binding. However, an increased maintenance dose of three tablets bid did not result in higher steady state concentrations compared to the normal maintenance dose of two tablets bid.

Similarly high plasma levels (26.5 μg/ml, IQR 18.9–31.5) after a double loading dose were reported in a large study by Marzolini et al., in which a different maintenance dosing scheme was used (Marzolini et al., 20202020). The SS plasma trough concentration in two studies with 21 and 11 COVID-19 patients who received ND LPV/RTV were 15.2 μg/ml (range 5.2–30.1 μg/ml) (Baldelli et al., 2020) and 18 μg/ml (range 11.4–30.8 μg/ml) (Gregoire et al., 2020) respectively, which is similar to our HD-SS and ND-SS results. The median unbound fraction of LPV in this study was 0.82% (range 0.38–1.52%) (Gregoire et al., 2020). LPV/RTV is known to be highly protein bound and only 1–2% of the drug is active and free (Croxtall and Perry, 2010) so even the HD LPV/RTV dosing scheme does not lead to sufficiently high plasma levels to inhibit SARS-CoV-2.

Interestingly, a 50% higher LPV drug exposure during maintenance therapy after day 2 did not result in higher plasma drug levels when compared to normal dose LPV therapy. One possible explanation could be the rapid reduction of inflammation as most patients in the HD-SS group were receiving dexamethasone as standard of care, whereas patients in the ND-SS were not. Additionally, dexamethasone is a known CYP3A4 inducer which contributes to the metabolization of LPV (McCune et al., 2000). Both, the anti-inflammatory and CYP3A4-inducing effect of dexamethasone did lower the LPV concentration in the high dose group. In fact, C-reactive protein was much lower in patients’ samples for HD-SS vs. ND-SS. Similarly, LPV plasma levels were found to be lower in patients who received the anti-inflammatory drug tocilizumab, but none of the patients in this study were prescribed corticosteroids (Marzolini et al., 2020).

In HIV patients, in whom significant hyperinflammation is rare, LPV plasma levels range from 4.9–7.1 μg/ml and are lower than in COVID-19 patients (Boffito et al., 2003; Croxtall and Perry, 2010; Baldelli et al., 2020). LPV is highly metabolized by the liver and cytochrome P450 3A4 enzymes (Croxtall and Perry, 2010) and it has been shown that these enzymes are downregulated during inflammation (Morgan, 2009; Schoergenhofer et al., 2018). Inflammation-induced downregulation of cytochrome P450 could be a possible explanation for the higher plasma concentration in COVID-19 patients. This hypothesis is supported by the positive correlation between C-reactive protein and the HD-SS and ND-SS plasma levels in our cohort. Another study showed the same positive correlation (r = 0.37, *p* < 0.001) in COVID-19 patients (Marzolini et al., 2020).

There was a trend towards a higher LPV concentration in females, while RTV plasma levels were significantly higher in females. This phenomenon was also reported in HIV patients. The reasons are not fully elucidated; differences in body weight, volume of distribution, drug-drug interactions or differences in transporter or enzyme expression have been discussed (Umeh et al., 2011).

SARS-CoV-2 replicates primarily in the respiratory tract (Sanders et al., 2020), so the drug concentration in the epithelial lung fluid (ELF) seems to be a more suitable parameter than the plasma level. In a study with HIV patients the LPV ELF concentration was 14.4 μg/ml while the plasma concentration was 8.1 μg/ml, which suggests that LPV accumulates in the ELF (ELF/plasma ratio = 1.78) (Atzori et al., 2003). Even if we assume that the LPV concentration in the ELF is twice as high as in plasma, the concentration would still be too low to inhibit SARS-CoV-2 sufficiently. To the best of our knowledge no study has determined the LPV ELF concentration in COVID-19 patients so far.

Interestingly, LPV has an EC50 of 4.1 μg/ml for SARS-CoV-1 and EC50 of 10.8 μg/ml for MERS which are lower compared to SARS-CoV-2 (Sanders et al., 2020). As LPV/RTV is used for the treatment of HIV, it is not surprising that in this case the EC50 is low (0.07 μg/ml) and can be readily achieved in plasma (Croxtall and Perry, 2010).

As previously mentioned, none of the large RCTs showed any beneficial effect of LPV/RTV treatment on major clinical outcome parameters (Cao et al., 2020; Collaborative Gr, 2020; Pan et al., 2021). While LPV/RTV is often used in combination with ribavirin and interferon to treat infections with SARS-CoV-1 (Chan et al., 2003; Chu et al., 2004) and MERS (Choi et al., 2016), it was rarely combined with those drugs to treat COVID-19 (Cao et al., 2020; Collaborative Gr, 2020; Pan et al., 2021). Triple therapy was associated with reduced viral shedding and reduced symptom burden in one trial when administered early in the COVID-19 disease course. As those patients had mild to moderate disease, the effect on mortality could not be assessed (Hung et al., 2020). The combination therapy might be effective in reducing clinically important outcome parameters. When our trial was started, we did not consider combination therapy with ribavirin and interferon as an option because our aim was to analyze the effect of high dose LPV/RTV by its own. Combination therapy would have led to more confounding and maybe an additional risk for side effects.

Side effects were common in our study. While the rate of diarrhea and nausea were comparable to other studies in COVID-19 patients, increased liver enzymes were more common in our population (Gregoire et al., 2020; Hung et al., 2020; Karolyi et al., 2020). Remarkably, patients with a LPV PLD level above median had a higher incidence of ALAT ≥5 ULN, which indicates a dose specific relationship. This limits the maximum dosage and a further increase is likely to result in harm to patients.

The strength of our study is that we used a unique dosing scheme which has not been used in any other COVID-19 study. Additionally, we measured plasma trough levels twice in our patients, when possible. Furthermore, we compared the HD plasma concentrations with ND from a previous cohort.

Our study also demonstrates limitations. The plasma samples were taken during routine daily blood sampling, so we were not able to collect PLD and SS samples in all patients for various reasons. For example, if patients were transferred to the ICU, treatment with LPV/RTV was stopped and plasma levels not monitored anymore. Further reasons included wrong tubes for plasma sampling or broken tubes during transport. For a detailed description see Supplementary Figure S1. Moreover, the sample size of patients who received ND LPV/RTV is small, we did not collect samples from the ELF and we only measured the total LPV/RTV concentration and not the free unbound fraction. No patient in the ND group received dexamethasone treatment because at that time anti-inflammatory treatment was not considered standard of care, which is a confounding factor.

In summary, after a double loading dose and 50% higher than normal LPV maintenance dose, plasma drug levels exceeded those of HIV patients by a factor of 2–5, most likely due to inflammation-induced cytochrome P450 downregulation. However, due to the high EC50 of LPV/RTV for SARS-CoV-2 and the fact that this drug is highly protein bound, high-dose LPV/RTV does not reach significant plasma levels and it seems unlikely that LPV/RTV exhibits any antiviral effect *in vivo* in COVID-19 patients, particularly in light of adjunctive anti-inflammatory treatment. Due to those reasons and the dose-dependent hepatotoxicity of LPV/RTV, higher doses should not be used in further studies.

## Data Availability

The raw data supporting the conclusion of this article will be made available by the authors, without undue reservation.
