# Supplementary material for: High Dose Lopinavir/Ritonavir Does Not Lead to Sufficient Plasma Levels to Inhibit SARS-CoV-2 in Hospitalized Patients With COVID-19
Source: Front Pharmacol. 2021 Jul 1;12:704767. doi: 10.3389/fphar.2021.704767 (PMC8282360; doi:10.3389/fphar.2021.704767)
Supplement: Supplementary file 1 [file DataSheet1.PDF]

Supplemental

Figure S1 – study flow diagram

High dose LPV/RTV plasma trough levels were measured (N=50)

Normal dose LPV/RTV plasma trough levels were measured during steady state (N=8)

PLD LPV/RTV was measured (n=43)

7 patients did not have a PLD measured:

- 3 patients did not get PLD because of daily routine practices
- 2 tubes broke
- 1 patient did not take the loading dose properly
- 1 wrong tube was used for drawing blood

SS LPV/RTV was measured (n=33)

17 patients did not have SS measured:

- 5 patients did not get PLD because of daily routine practices
- 4 patient were transferred to the ICU and no SS was taken
- 2 tubes broke
- 2 patient were discharged
- 2 wrong tubes were used for drawing blood
- 1 sample was not properly labeled
- 1 patient quit study drug due to tablet size

Abbreviations: LPV=lopinavir, RTV=ritonavir, PLD=post-loading dose, SS=steady state
